# Supplementary figures and images for: Gene expression in Tribolium castaneum life stages: Identifying a species-specific target for pest control applications
Source: PeerJ. 2019 May 23;7:e6946. doi: 10.7717/peerj.6946 (PMC6535216; doi:10.7717/peerj.6946)

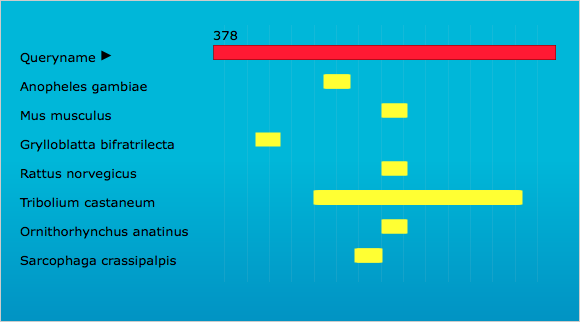

Supplement: Figure S1 — The red bar indicates the sequence of T. castaneum LOC103313766 CPG, and the yellow bars show potential sequence regions susceptible to off target effects in the corresponding species. [file peerj-07-6946-s001.png]

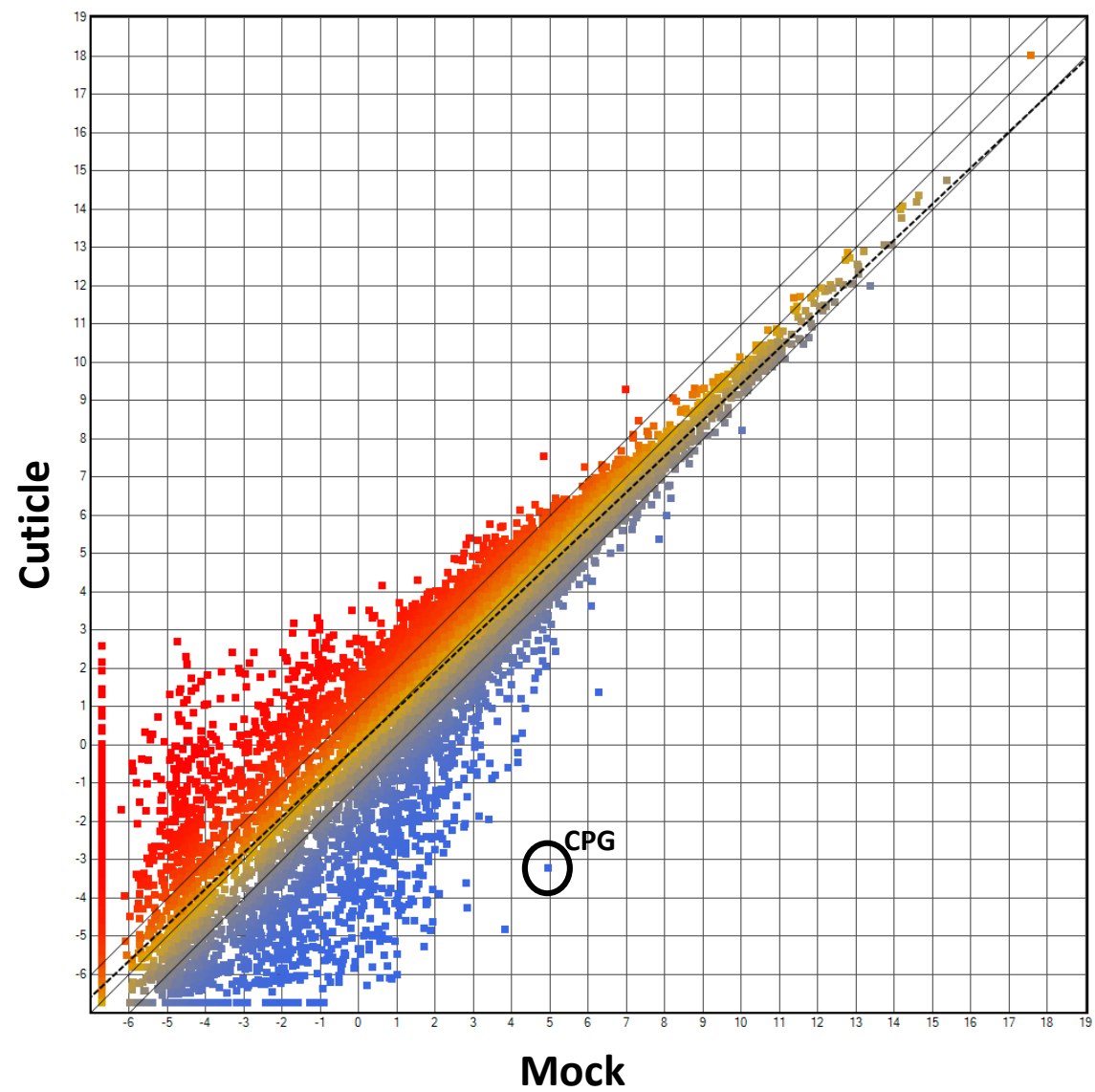

Supplement: Figure S2 — The scatterplot shows the differential expression of genes between Mock and CPG RNAi treated larvae. The x-axis is gene expression (RPKM) for Mock and the y-axis is gene expression (RPKM) for CPG RNAi treated larvae. The solid lines indicate the upper lower 95% confidence interval and the dotted line denotes the linear correlation between Mock and CPG RNAi. CPG is labeled and circled. [file peerj-07-6946-s002.pdf]
