# Supplementary material for: Gene expression in Tribolium castaneum life stages: Identifying a species-specific target for pest control applications
Source: PeerJ. 2019 May 23;7:e6946. doi: 10.7717/peerj.6946 (PMC6535216; doi:10.7717/peerj.6946)
Supplement: File S5 — Total reads refer to the number of useable reads. [file peerj-07-6946-s007.docx]

Supplementary File 5. Illumina MiSeq sequencing metrics for biological and technical replicates of treatment (RNAi knockdown of LOC103313766) and controls (Mock and Control) in *T. castnaeum* larvae. Total reads refers to the number of useable reads.

| **Treatment/Control** | **Biological Replicate** | **Technical Replicate** | **Total reads** |
| --- | --- | --- | --- |
| LOC103313766 dsRNA | 1 | a | 6,648,806 |
| LOC103313766 dsRNA | 1 | b | 3,901,980 |
| LOC103313766 dsRNA | 1 | c | 4,545,890 |
| LOC103313766 dsRNA | 1 | d | 4,166,760 |
| LOC103313766 dsRNA | 2 | a | 4,502,510 |
| LOC103313766 dsRNA | 2 | b | 4,115,930 |
| LOC103313766 dsRNA | 2 | c | 4,256,296 |
| LOC103313766 dsRNA | 2 | d | 2,395,838 |
| LOC103313766 dsRNA | 3 | a | 4,027,778 |
| LOC103313766 dsRNA | 3 | b | 4,166,760 |
| LOC103313766 dsRNA | 3 | c | 4,462,068 |
|  |  |  |  |
| Control | 1 |  | 4,097,238 |
| Control | 2 |  | 4,195,076 |
| Control | 3 |  | 3,736,774 |
|  |  |  |  |
| Mock | 1 |  | 3,259,586 |
| Mock | 2 |  | 5,025,754 |
| Mock | 3 |  | 3,124,730 |
